# Development, Reliability and Validity of Engagement in Exercise Rehabilitation Scale for Patients with Stroke

**DOI:** 10.3390/nursrep15080303

**Published:** 2025-08-19

**Authors:** Hu Jiang, Xiaoxuan Wang, Wenna Wang, Yongxia Mei, Beilei Lin, Jing Chen, Zhenxiang Zhang

**Affiliations:** 1 School of Nursing and Health, Zhengzhou University, Zhengzhou 450001, China; jianghuchn@163.com (H.J.); wangxiaoxuan222666@163.com (X.W.); wwn15290817551@163.com (W.W.); linbeilei@zzu.edu.cn (B.L.); 2 Nursing Department, The Third Affiliated Hospital of Zunyi Medical University, The First People’s Hospital of Zunyi, Zunyi 563000, China

**Keywords:** stroke, patient engagement, exercise, rehabilitation, scale, reliability, validity

## Abstract

**Background**: Exercise rehabilitation is a crucial component of stroke recovery, particularly for patients transitioning to home or community settings. However, there is currently a lack of self-reported scales designed to measure the level of engagement in exercise rehabilitation among patients with stroke. **Objective**: To develop and validate psychometric properties of the Engagement in Exercise Rehabilitation Scale for patients with stroke. **Methods**: The initial item pool was extracted from a literature review and a semi-structured interview with patients with stroke. The development and refinement of the items underwent expert consultation and cognitive interviews with patients with stroke. The items primarily covered patients’ perceptions, emotional attitudes, and specific engagement behaviors regarding exercise rehabilitation in home or community settings. A total of 260 patients with stroke were selected to test the reliability and validity. The psychometric proprieties test included construct validity, content validity, criterion-related validity, exploratory factor analysis, internal consistency reliability, test–retest reliability, and split-half reliability. **Results**: The final version of the Engagement in Exercise Rehabilitation Scale comprised 20 items. The scale’s content validity index was determined to be 0.976, while the item-content validity indices ranged from 0.833 to 1.000. Results from exploratory factor analysis indicated that this scale is unidimensional, with a cumulative variance contribution rate of 79.3%. The test–retest reliability of the scale was found to be 0.879, its split-half reliability was measured at 0.980, and its Cronbach’s α coefficient was calculated to be 0.986. **Conclusion**: The Engagement in Exercise Rehabilitation Scale for patients with stroke demonstrates accepted reliability and validity. The accuracy and generalizability of this scale necessitate further validation through additional large-sample studies involving diverse populations across multiple centers.

## 1. Introduction

Stroke is one of the most prevalent chronic non-communicable diseases, characterized by its high incidence rate, high recurrence rates, substantial disability prevalence, high mortality, and substantial economic burden [1]. Currently, stroke ranks as the second leading cause of death and the third leading cause of disability globally [2]. It is also recognized as the primary cause of long-term neurological disability worldwide [3], with approximately 80% of patients with stroke experiencing limb dysfunction. Furthermore, 15–30% suffer from severe disabilities that result in a significant decline in independent living ability and compromised quality of life [2]. At least 50% of stroke survivors become disabled due to motor impairments in either or both upper and lower limbs [4]. The disabilities or dysfunctions caused by stroke necessitate prolonged rehabilitation, imposing a significant long-term burden on healthcare systems. These challenges require urgent attention from governments and healthcare providers worldwide.

Following a stroke, rehabilitation serves as an effective strategy to reduce disability rates, promote recovery, enhance independence, and improve overall quality of life [5,6]. Exercise rehabilitation remains a crucial component in the recovery process for patients with stroke due to the high prevalence of functional deficits associated with this condition [5]. This form of rehabilitation encompasses all types of exercise and physical activity aimed at enhancing both physical and mental functions, as well as facilitating participation in daily activities [7,8,9]. Adherence to exercise rehabilitation offers numerous benefits. It not only aids in the restoration of patients’ physical motor functions but also enhances cognitive abilities, mitigates cardiovascular risks, improves quality of life, and supports patients’ reintegration into society or the work [10,11,12]. Although early implementation of rehabilitation has been shown to significantly improve clinical outcomes for patients [13], some studies have indicated that engagement levels are often suboptimal [14,15]. In recent years, researchers have increasingly highlighted the critical importance of patient engagement in the rehabilitation process [16,17].

Patient engagement is an emerging concept that has become a focal point in contemporary healthcare [18]. It underscores the notion that patients are cocreators of their own health, highlighting the significance of their proactivity and active collaboration [19]. The rehabilitation process following a stroke is lengthy, necessitating sustained effort over several months or even years. Patient engagement during this rehabilitation phase plays a crucial role in optimizing outcomes [20]. Building on previous concepts related to patient engagement in rehabilitation [21,22,23,24], our analysis defines patient engagement in this context as a dynamic and evolving process [25]. In this process, patients are expected to maintain an optimistic outlook, demonstrate deliberate commitment, and invest persistently while actively communicating and collaborating throughout their rehabilitation journey.

For the majority of patients with stroke, home or community rehabilitation currently serves as the primary option [26,27]. More than 50% of these patients transition directly to home for rehabilitation following acute phase treatment in a hospital setting [28,29,30]. Additionally, home- or community-based rehabilitation has emerged as a new trend for patients with stroke due to advancements in technology and modifications to healthcare models [31,32,33]. Consequently, exploring engagement in exercise rehabilitation for patients with stroke within home or community environments represents a significant area for further research.

To date, measurement tools related to patient engagement in rehabilitation remain underdeveloped. Among patients with stroke, the assessment instruments for evaluating patient engagement in rehabilitation predominantly rely on professional assessments, such as the Hopkins Rehabilitation Engagement Rating Scale (HRERS) [22], the Pittsburgh rehabilitation participation scale (PRPS) [34], the occupational therapy engagement scale (OTES) [24], and Rehabilitation Therapy Engagement Scale (RTES) [35]. These tools primarily emphasize engagement in rehabilitation therapy rather than specifically addressing exercise rehabilitation. Moreover, their application at home or within community settings is significantly hindered by a lack of available healthcare professionals. In contrast, self-rated scales that depend on patient self-reports [36] can quantitatively measure levels of patient engagement in exercise rehabilitation, rendering them more suitable for use in community or home environments. Additionally, it is important to note that the concept and attributes of patient engagement in rehabilitation may differ based on the content and context of rehabilitative practices; thus, the aforementioned measurement tools may not be fully applicable across all scenarios [25].

Patient engagement in exercise rehabilitation is positively associated with health outcomes and quality of life [25]. However, the current absence of specific assessment tools impedes researchers and clinicians from obtaining a comprehensive understanding of patients’ engagement in home- or community-based exercise rehabilitation. There is an urgent need to develop a new measurement instrument that addresses the limitations of existing rehabilitation engagement scales concerning content, feasibility, and relevance to patient experience. Therefore, the objective of this study was to develop and validate the psychometric properties of the Engagement in Exercise Rehabilitation Scale (EERS) for patients with stroke.

## 2. Methods

### 2.1. Conceptual Framework

This study employed the Model for Therapeutic Engagement in Rehabilitation, as proposed by Lequerica et al. [21], as its theoretical framework. This model elucidates the factors that influence patients’ willingness to engage in rehabilitation treatment and outlines their developmental process, highlighting the significance of both personal and environmental variables in facilitating patient participation. Key determinants affecting rehabilitation engagement encompass patients’ perceived needs, anticipated outcomes, self-efficacy, and physical condition. The engagement process can be categorized into distinct stages: intention formation, preparation, active engagement, and experience evaluation. We defined patients’ engagement in exercise rehabilitation as characterized by a hopeful attitude, well-considered commitments, sustained engagement, and proactive communication and collaboration with rehabilitation providers. This approach ensures that patients can continuously adapt to the exercise rehabilitation plan while striving to achieve their individual rehabilitation goals [25].

**Figure 1:**
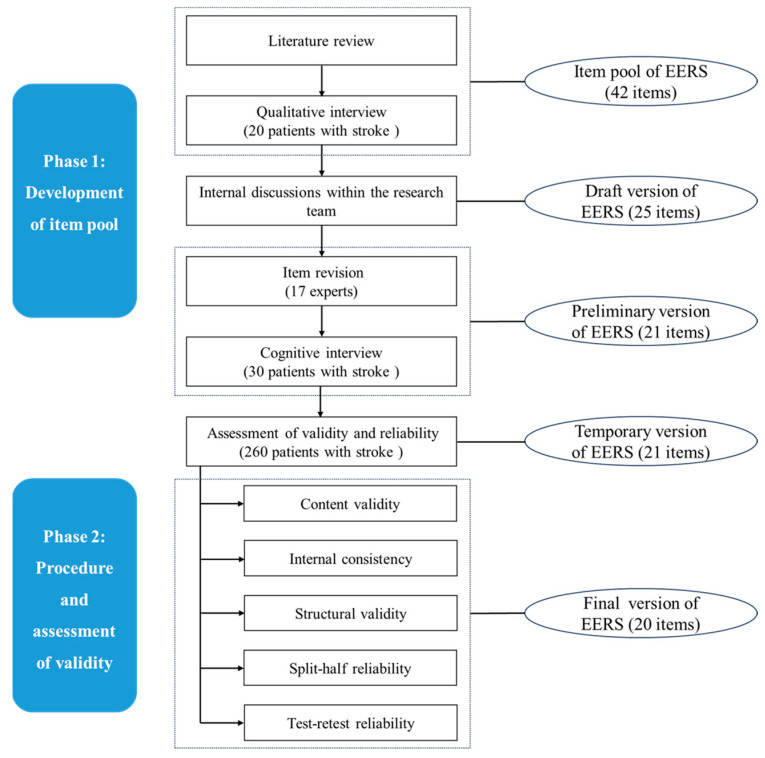
The development process of EERS. Note: EERS, the Engagement in Exercise Rehabilitation Scale.

In this study, the construction of the scale mainly included two phases. The development process is shown in Figure 1.

### 2.2. Phase 1: Development of Item Pool

#### 2.2.1. Literature Review

Firstly, a literature review was conducted. Databases including PubMed, Web of Science, Scopus, Embase, APA PsycINFO, and CINAHL were systematically searched. The search period extended from the inception of these databases up to 31 March 2024. Search terms related to “stroke,” “rehabilitation,” “engagement,” and “scale” were employed. A total of 3729 documents were retrieved across the six databases. Among these, 1847 duplicates were removed both manually and using EndNote X9.1 software. Following an initial screening of titles and abstracts, an additional 1848 documents were excluded. Through a thorough examination of full texts, ten documents ultimately met the inclusion criteria (the results are presented in the Supplementary Materials). Based on the literature analysis conducted by our research team, twenty items were developed from the reviewed studies.

#### 2.2.2. Qualitative Interview

This study explored the attitudes, perceptions, and lived experiences of community-based stroke survivors toward exercise rehabilitation through in-person, semi-structured interviews. The purposeful sampling method was employed to recruit participants, resulting in a total of 20 patients from the outpatient department of a general hospital and a community who participated in this study. The outline of the interview included the following questions: ① Could you share your understanding of exercise rehabilitation? ② Could you talk about how you got engaged in exercise-based rehabilitation at home? ③ Could you share your experiences and feelings during the home-based rehabilitation process? The findings from this section have been published elsewhere [37]. In line with the principles of descriptive qualitative research, 22 items were generated based on patient interviews.

#### 2.2.3. Formulation of the Draft EERS

Based on the aforementioned two components of the study, a total of 42 items were developed. Taking into full account the cultural context and linguistic habits prevalent in China, a preliminary screening and integration of the item pool was conducted. Following discussions among members of the research team, 12 items that were not relevant to the theme were eliminated, while 10 redundant items were consolidated into 5 distinct items. Ultimately, it was decided to include 25 items in the initial scale.

#### 2.2.4. Item Revision

To further enhance the content validity of the scale, the expert consultation method was employed, where experts judged the importance and relevance of the items and provided corresponding suggestions. The selection criteria for experts were as follows: ① Engaged in or studied in the field of chronic disease care, community nursing, clinical medicine, rehabilitation medicine, etc., for more than 10 years; ② Possessing an associate senior title or above and a bachelor’s degree or higher; ③ Experienced in stroke rehabilitation or scale development; ④ Voluntary involvement and informed consent. Exclusion criteria were as follows: Those who cannot return the questionnaire on time. The consultation form mainly includes an explanation of the consultation, a survey of the general information, familiarity, and basis of judgment of experts, and a rating of item importance and item relevance. Experts self-assess their familiarity with the indicators and the basis of their judgment. The importance of the items is rated using a 5-point Likert scale, ranging from “very unimportant” to “very important” with scores from 1 to 5; relevance evaluation ranges from “very irrelevant” to “very relevant” with scores from 1 to 5. The criteria for item selection are an item importance average score of ≥4, a content validity index of ≥0.78, and a coefficient of variation of <0.25 [38].

Two rounds of consultations were conducted from May to July 2024. In the first round, 20 questionnaires were distributed, yielding 17 valid responses and an expert participation rate of 85%. In the second round, 17 questionnaires were sent out, resulting in 15 valid responses and an expert participation rate of 88.2%.

Following these two rounds of expert inquiries, Kendall’s coefficients of concordance were calculated at 0.103 and 0.112 (both *p* < 0.05), with corresponding expert authority coefficients of 0.92 and 0.91, respectively. The importance scores from the first round ranged from 4.24 to 5.00, exhibiting a coefficient of variation between 0 and 0.197. Results were summarized based on item selection criteria and expert recommendations; three items were removed, language descriptions for nine items were revised, and two items were merged. In the second round of inquiries, importance scores ranged from 4.73 to 5.00 with a coefficient of variation between 0 and 0.096; six items underwent modifications during this phase as well. Items continued to be merged, deleted, or modified according to expert feedback until consensus was reached after both consultation rounds concluded, culminating in a preliminary draft scale. The final revised scale comprises a total of 21 items.

#### 2.2.5. Cognitive Interview

Taking into account the variations in the research subjects’ place of residence, educational background, age, and other relevant factors, a convenience sample of 30 patients with stroke residing in the community was selected to conduct cognitive interviews on each item of the scale. This process aimed to observe their comprehension of the items and their response selection process, with the goal of refining the clarity of wording for each item, enhancing readability, and improving the accuracy of formal surveys. Following this testing phase, it was found that the average time required to complete a single questionnaire ranged from 4 to 8 min. During this stage, no feedback was solicited from patients, nor were any modifications made to the scale items, thus resulting in what is considered the initial version of the scale.

### 2.3. Phase 2: Procedure and Assessment of Validity

#### 2.3.1. Sample Size

According to factor analysis, the sample size should be no less than 5 to 10 times the number of items [39]. Taking into account a potential 10% rate of invalid questionnaires, the estimated maximum required sample size is 242 cases. Since the actual sample size exceeds 200, it is deemed acceptable for exploratory factor analysis (EFA) [40].

#### 2.3.2. Participants

The subjects with a history of stroke were recruited for this study, which was conducted across five communities in Pingdingshan City, Henan Province. The inclusion criteria were as follows: ① individuals aged 18 years or older; ② those who met the diagnostic criteria for stroke [41]; ③ confirmed by CT or MRI imaging; ④ possessing a disability level below moderate (modified Rankin Scale (mRS) score ≤ 3); ⑤ demonstrating normal language abilities (Token Test score ≥ 17) and cognitive function (Mini-Mental State Examination, MMSE score ≥ 27); ⑥ capable of responding to questions accurately and coherently; ⑦ in the non-acute phase of recovery (onset of stroke > 7 days) [41]; ⑧ discharged from hospital and residing within the community; and ⑨ providing informed consent to participate in the study. Individuals with other serious medical conditions, such as malignant tumors, heart failure, respiratory failure, or severe injuries, along with those concurrently enrolled in other research programs, were excluded based on specific criteria.

#### 2.3.3. Measurements

General information questionnaire: The questionnaire was developed by the researchers and included items such as gender, age, education level, marital status, disease duration, stroke type, number of strokes, and other sociodemographic data and disease-related information.

Engagement in Exercise Rehabilitation Scale for Patients with Stroke (EERS): This version of the scale comprises 21 items. It employs a Likert 5-point scoring method where 1 point indicates “never,” 2 points indicate “rarely,” 3 points indicate “sometimes,” 4 points indicate “often,” and 5 points indicate “always.” A higher score reflects a greater degree of patient engagement in exercise rehabilitation.

Functional exercise compliance scale for community patients with stroke: This scale was previously developed by the research team [42] and demonstrates a content validity index of 0.95. It has an internal consistency reliability measured by Cronbach’s α coefficient of 0.90 and test–retest reliability intraclass correlation coefficients exceeding 0.70, indicating strong reliability and validity that align well with the objectives of this study. The scale consists of three dimensions encompassing a total of 14 items. Each item is rated on a four-point scale ranging from 1 to 4. To facilitate comparison across subjects, compliance scores are converted into a compliance index calculated as follows: Compliance Index = (Actual Compliance Score/Theoretical Maximum Compliance Score) × 100. A higher compliance index signifies greater adherence to rehabilitation exercises. In our study, this scale served as the criterion measure.

#### 2.3.4. Data Collection

Two trained investigators screened participants who met the inclusion criteria based on their medical records and provided an explanation of the study’s purpose and significance to the research subjects, in accordance with the principles of voluntariness, anonymity, and confidentiality. Upon obtaining informed consent from the research subjects to participate in the study, paper questionnaires were distributed. Data collection occurred from July to October 2024. After completion of the questionnaires, the investigators verified their completeness through a thorough review process. Test–retest reliability was assessed two weeks following the initial measurement wave, with 30 cases randomly selected using Excel’s random number generation capabilities.

### 2.4. Ethical Considerations

The study was approved by the Ethics Committee of Zhengzhou University (ZZUIRB 2023-279) on 10 October 2023. All study subjects agreed to participate voluntarily and signed the informed consent form.

### 2.5. Statistical Analysis

Data analysis was performed using SPSS version 26.0. Descriptive statistics were employed to summarize the basic characteristics of the research subjects. A *p*-value of less than 0.05 was considered indicative of a statistically significant difference. Differences between subgroups were analyzed using independent samples *t*-tests and one-way ANOVA.

Item screening utilized three psychometric validation methods. Critical ratio analysis was employed to evaluate item discrimination. Participants were ranked based on their total scale scores, with the top 27% and bottom 27% of patients with stroke designated as the high-performance and low-performance groups, respectively [43]. Independent samples *t*-tests were conducted to compare mean scores for each item between these two groups. Items that did not demonstrate significant discrimination (*p* > 0.05 or critical ratio < 3.0) were excluded from further consideration. Item-total correlation was used to assess the degree to which each item correlated with the overall scale score. Items exhibiting non-significant or weak correlations (*r* < 0.30) were discarded [44]. Additionally, Cronbach’s α optimization was performed to analyze internal consistency reliability [45]. Items whose removal resulted in an increase in the scale’s α coefficient were considered for elimination.

Content validity was assessed by experts utilizing a 4-point Likert scale (1 = not relevant to 4 = very relevant). The item-content validity index (I-CVI) was computed as the proportion of experts rating an item at least 3. The scale-content validity index (S-CVI) represents the mean I-CVI across all items, with thresholds set at I-CVI ≥ 0.78 and S-CVI ≥ 0.90 [38]. Construct validity was evaluated through exploratory factor analysis (EFA), which involved calculating the correlation matrix between each dimension and the total score of the scale, alongside conducting internal correlation analyses. The appropriateness for EFA was determined using the Kaiser–Meyer–Olkin (KMO) measure of sampling adequacy; a KMO value below 0.6 indicated unsuitability for EFA, while Bartlett’s test of sphericity required a significance level of *p* < 0.01 [46]. When conditions for EFA were satisfied, the number of common factors was identified using varimax rotation. Criteria for item selection included eigenvalues greater than 1 and factor loadings exceeding 0.4, with a cumulative contribution rate from common factors above 40% [47]. Criterion-related validity was examined by calculating the correlation coefficient between scale scores and those from the Functional Exercise Compliance Scale among community patients with stroke, thereby assessing criterion-related validity for this scale.

Three reliability measures were employed to evaluate the questionnaire’s psychometric properties: internal consistency, test–retest reliability, and split-half reliability [48]. Internal consistency was assessed using Cronbach’s α coefficient, where values >0.7 indicate acceptable scale homogeneity [49]. Test–retest reliability was examined through Pearson correlation analysis of scores from two administrations, with coefficients >0.7 considered satisfactory [50]. Split-half reliability was determined via the odd-even method, whereby items were divided into two subsets, and their score correlation was computed.

## 3. Results

### 3.1. Characteristics of Participants

A total of 285 questionnaires were distributed, with 260 completed and returned, yielding an effective response rate of 93.4%. The average age of the research participants was (66.46 ± 9.75) years; among them, there were 191 males and 69 females. More than half of the patients had experienced strokes more than twice, comprising 238 cases of ischemic stroke, 6 cases of hemorrhagic stroke, and 16 cases of mixed stroke. The basic demographic information of the participants is presented in Table 1.

### 3.2. Item Analysis

According to the findings derived from the critical ratio approach, the differences between high and low groups for each item were statistically significant (*p* < 0.05). The results obtained from the correlation coefficient method indicated a strong overall correlation between individual items and the total scale, with correlation coefficients ranging from 0.423 to 0.930. The Cronbach’s α coefficient for the entire scale was calculated to be 0.984, and removing any single item did not lead to an increase in this coefficient, suggesting that all items contribute meaningfully to the internal consistency of the total scale. Consequently, none of the items on the original scale were excluded based on these three analytical approaches.

### 3.3. Validity

Conveniently, a panel of 10 experts was selected to conduct a face content evaluation. Out of these, eight experts completed the assessment, and the mean importance scores for each item exceeded 4.0 points. The item-content validity index (I-CVI) ranged from 0.88 to 1.00, while the scale-content validity index (S-CVI) was calculated at 0.976, indicating strong content validity.

The results of Bartlett’s test of sphericity yielded a χ^2^ value of 7877.174 (*p* < 0.001), and the KMO value was determined to be 0.977, suggesting that the data were appropriate for factor analysis. The measures of sampling adequacy for each item varied from 0.170 to 0.882; notably, only item 21 had a score below the acceptable threshold of 0.500.

Using principal component analysis with a promax rotation, the initial round of EFA revealed that two common factors exhibited eigenvalues greater than 1, accounting for a cumulative variance contribution rate of 76.3%. The scree plot indicated that the slope began to flatten after the second factor. Notably, only item 21 constituted the second common factor, which had an eigenvalue of 0.413.

Following internal discussions, this item was removed from consideration, and further analyses were conducted. In the subsequent round of EFA, one common factor was extracted. Bartlett’s test of sphericity yielded a χ^2^ value of 7748.391 (*p* < 0.001), while the Kaiser–Meyer–Olkin (KMO) measure resulted in a value of 0.980. The cumulative variance contribution rate for this common factor reached 79.3%, with all items demonstrating loadings exceeding 0.8 on their respective factors. The results pertaining to factor loading are presented in Table 2.

### 3.4. Reliability

After conducting a Spearman correlation analysis, the correlation coefficient be-tween the EERS and the Functional Exercise Compliance Scale was found to be 0.925 (*p* < 0.01), indicating a high level of criterion-related validity. The overall Cronbach’s α coefficient was calculated to be 0.986 (95% CI: 0.984–0.988), while the total split-half reliability was determined to be 0.980, demonstrating good internal consistency.

Two weeks following the initial distribution of the questionnaire, a subset of 30 participants was selected from among the 260 respondents for a resurvey. The characteristics of these participants are detailed in Table 1. The test–retest reliability for the entire scale was measured at 0.879, suggesting that the scale possesses good stability.

### 3.5. Analysis of Demographic Data

We further conducted subgroup analyses to examine the effects of gender and stroke type on EERS scores, with results presented in Table 3.

## 4. Discussion

This study developed and validated a 20-item scale designed to assess the engagement level in exercise rehabilitation among patients with stroke. The findings demonstrated satisfactory content validity, internal consistency, structural validity, split-half reliability, and test–retest reliability.

This study introduced a novel scale specifically designed to assess engagement in home- or community-based rehabilitation among patients with stroke. More precisely, the scale primarily emphasizes engagement in exercise rehabilitation, thereby distinguishing it from existing scales that predominantly evaluate patient engagement during in hospital rehabilitation therapy [16]. Accumulated evidence supports the efficacy of exercise therapy as a validated method for alleviating common sequelae associated with stroke [51]. Furthermore, home-based rehabilitation has proven to be an effective strategy for enhancing quality of life and reducing disability among stroke survivors [52]. A recent study [53] investigating stroke survivors’ preferences regarding self-reported outcomes indicated that 86.25% of participants preferred completing a written survey. This research addresses the current gap in measurement tools aimed at evaluating patient engagement in exercise rehabilitation for patients with stroke residing in home or community settings.

In the process of scale development, this study adhered rigorously to established principles and procedures for scale development [45]. The theoretical framework employed was based on the model for therapeutic engagement in rehabilitation [21]. Additionally, a comprehensive literature review and qualitative research provided a robust theoretical foundation essential for developing an item pool. This study adopted the Likert 5-point scale, as it is one of the most commonly used rating scale [54]. Following initial modifications to the items, the study ultimately yielded a 21-item scale for investigation. Through two rounds of EFA, one item was eliminated from consideration. The 21st item that was removed primarily addressed the necessity for patients to receive family assistance during exercise rehabilitation. However, most surveyed patients exhibited a certain degree of mobility, which resulted in generally low scores for this particular item. This discrepancy may also explain why it did not correlate well with other items within the same dimension. After thorough discussion, we concluded that when patients possess sufficient mobility, they are less likely to seek assistance from others; thus, we decided to exclude this item. After refinement, the final version of the scale comprised 20 items.

The findings of this study demonstrated strong validity and reliability for the scale. The psychometric properties of the scale were rigorously evaluated, including content validity, internal consistency, structural validity, split-half reliability, and test–retest reliability. All assessed parameters met the established criteria. The development of the scale was conducted in accordance with classical test theory principles without utilizing item response theory methodologies [55]. Importantly, all retained items exhibited very high loadings (>0.8), which may suggest a degree of similarity among items. Future research will involve an evaluation using the Rasch model with large-sample data to further validate and refine the scale items [56].

The results of this study indicate that the scale is unidimensional. Some studies [23,57] have suggested that engagement in rehabilitation encompasses three dimensions: cognitive, emotional, and behavioral; however, our study did not confirm this. Given the ambiguity surrounding the dimensions of patient engagement in exercise rehabilitation, we refrained from predefining these dimensions within the item pool. Nevertheless, our items included aspects related to cognition, behavior, and emotion. It is noteworthy that most existing scales [22,24,35] are also unidimensional, which aligns with our findings. Several factors may contribute to these observed discrepancies. Primarily at a theoretical level, current frameworks remain underdeveloped or lack consensus. Scales based on distinct theoretical foundations will inevitably exhibit variations in their dimensional structures. Although this study utilized a model for therapeutic engagement in rehabilitation [21], it does not explicitly delineate the attributes and dimensions of engagement. Another potential influencing factor pertains to the content of rehabilitation itself. Our research specifically focused on exercise rehabilitation; other domains may yield different dimensional configurations. Furthermore, population characteristics (e.g., cultural background and geographical location) among study participants may also result in dimensional inconsistencies across scales [58]. In summary, scale dimensionality arises from multiple interacting factors. While our current data support a unidimensional structure, further validation through additional studies is necessary to substantiate this finding.

The findings of this study indicate a positive association between patient engagement in exercise rehabilitation and exercise compliance. A conceptual analysis [59] suggests that therapeutic compliance is an integral aspect of engagement, while Hickmann et al. [60] also regard therapeutic compliance as a fundamental component of patient engagement. Given the conceptual overlap between compliance and engagement, exercise compliance was utilized as the criterion standard for validating the scale. To maximize the benefits derived from rehabilitation, it is essential for patients to actively engage in or invest effort into the rehabilitation process [21]. This scale demonstrates significant practical utility. Moving forward, emphasizing stroke patient engagement in exercise rehabilitation will be a critical focus within stroke management.

Subgroup analysis indicated that age may play a significant role in influencing patients’ engagement in exercise rehabilitation. However, no statistically significant differences were found across various stroke types or between genders. These findings suggest that patient engagement in rehabilitation differs among diverse populations, which also affects the reliability and validity of the results. Our previous concept analysis [25] demonstrated that patient engagement is shaped by multiple intrinsic and extrinsic factors. Due to our limited sample size, we were unable to perform comprehensive reliability and validity analyses across different subgroups. Given the heterogeneity of patients with stroke, we recommend that future studies validate the psychometric properties of the scale within varied populations.

This study offers several implications for future research. Firstly, additional investigations are necessary to more precisely define the attributes and dimensions of patients’ engagement in exercise rehabilitation, which would contribute to a clearer understanding of this concept. Furthermore, further validation studies could be conducted utilizing this scale to ascertain whether it is unidimensional or multidimensional. Moreover, future research should adopt qualitative, quantitative, or even mixed methods approaches to identify the factors that influence patients’ engagement in exercise rehabilitation [25]. Finally, researchers ought to explore the underlying mechanisms [61] and develop practical intervention strategies [62,63] aimed at enhancing rehabilitation outcomes and improving long-term quality of life for patients with stroke.

### Limitations

This study has several limitations that should be acknowledged. Firstly, all participants were recruited from Henan Province, China, which may not adequately represent the broader context of exercise rehabilitation engagement among patients with stroke nationwide. Secondly, this research utilized convenience sampling instead of stratified sampling. The majority of subjects in this study were male and had ischemic strokes, which could potentially affect the reliability and validity of the findings. Furthermore, due to constraints related to funding, manpower, and research time, a large-scale survey could not be conducted; consequently, no confirmatory factor analysis was performed.

## 5. Conclusions

This study developed a 20-item scale designed to assess the engagement level of exercise rehabilitation among patients who have experienced a stroke. The Exercise Engagement Rehabilitation Scale (EERS) was demonstrated to be unidimensional, as well as having satisfactory validity and reliability. This scale specifically addresses the needs of the increasing population of community-dwelling stroke survivors, providing a scientific and practical self-assessment tool for monitoring patient engagement in exercise rehabilitation. However, further validation is required across more diverse populations and various settings. Additionally, confirmatory factor analysis is necessary to reinforce the structural validity of the scale across different samples.

## Figures and Tables

**Table 1:** General characteristics of participants (*n* = 260).

Demographic Characteristics	Total Sample		Retest Group	
	*n*/Mean	%/SD	*n*/Mean	%/SD
**Age**	66.46	9.75	65.5	8.34
**Gender**				
Men	191	73.5	26	86.67
Women	69	26.5	4	13.33
**Education level**				
Middle school or lower	133	51.2	10	33.33
High school/technical secondary school	81	31.2	12	40.00
College or above	46	17.7	8	26.67
**Marital status**				
Married	227	87.3	28	93.33
Unmarried/Divorced/Widowed	33	12.7	2	6.67
**Number of strokes**				
1	115	44.2	14	46.67
2	86	33.1	9	30.00
3	41	15.8	5	16.67
≥4	18	6.9	2	6.67
**Type of stroke**				
Ischemic stroke	238	91.5	29	96.67
Hemorrhagic stroke	6	2.3	1	3.33
Mixed-type stroke	16	6.2	0	0.00
**Modified Rankin score**				
0	97	37.3	13	43.33
1	107	41.2	13	43.33
2	36	13.8	2	6.67
3	20	7.7	2	6.67

**Table 2:** Factor loading from EFA.

Item	Factor 1
1. I believe that exercise rehabilitation is a dynamic and continuous process that requires ongoing investment.	0.913
2. I have some knowledge and skills related to exercise rehabilitation.	0.840
3. I have clear goals for my exercise rehabilitation.	0.893
4. I can correctly evaluate the effectiveness of exercise rehabilitation.	0.899
5. I can apply the knowledge I have learned in exercise rehabilitation to real-life situations.	0.894
6. I can recognize my strengths and weaknesses in the exercise rehabilitation process.	0.892
7. I can make timely decisions to optimize my exercise rehabilitation plan.	0.909
8. I am willing to invest time and energy in exercise rehabilitation.	0.931
9. I have a positive attitude towards participating in exercise rehabilitation.	0.940
10. I can effectively manage negative emotions during the exercise rehabilitation process.	0.864
11. I feel proud and satisfied with the progress I make in the exercise rehabilitation process.	0.908
12. During the exercise rehabilitation process, I feel understood and supported by others (medical staff, family members, etc.).	0.825
13. I actively participate in the formulation of the exercise rehabilitation plan.	0.886
14. I have a regular exercise habit.	0.938
15. I interact and collaborate effectively with medical staff regarding exercise rehabilitation.	0.764
16. I learn methods and skills for exercise rehabilitation.	0.847
17. I concentrate on completing each exercise rehabilitation session.	0.924
18. I complete my exercise rehabilitation plan on time.	0.939
19. I adjust the intensity or content of exercise rehabilitation according to my own condition.	0.908
20. I monitor my exercise data independently.	0.884

Note: EFA, exploratory factor analysis.

**Table 3:** Differences in total EERS scores among subgroups.

Demographic Characteristics	Mean	SD	Statistic	*p*
**Age**			2.247	0.026
<65	59.34	21.25		
≥65	53.26	21.30		
**Gender**			0.295	0.864
Men	55.76	21.83		
Women	55.25	20.48		
**Type of stroke**			0.164	0.849
Ischemic stroke	55.85	21.66		
Hemorrhagic stroke	52.17	21.40		
Mixed-type stroke	53.56	19.09		

## Data Availability

The raw data will be available from the corresponding authors on reasonable request.

## Supplementary Materials

The following supporting information can be downloaded at: https://www.mdpi.com/article/10.3390/nursrep15080303/s1, Figure S1: Literature screening flow diagram; Table S1: Search strategy.

## Public Involvement Statement

No public involvement in any aspect of this research.

## Guidelines and Standards Statement

This study was designed and reported in accordance with the guidelines for Strengthening the Reporting of Observational Studies in Epidemiology.
