# Supplementary material for: Development, Reliability and Validity of Engagement in Exercise Rehabilitation Scale for Patients with Stroke
Source: Nurs Rep. 2025 Aug 19;15(8):303. doi: 10.3390/nursrep15080303 (PMC12388844; doi:10.3390/nursrep15080303)
Supplement: Supplementary file 1 [file nursrep-15-00303-s001.zip › nursrep-3738842-supplementary.pdf]

**Table S1.** Search strategy.

| Step | Search strategy |
|---|---|
| #1 | stroke OR “ischemic attack” OR “transient ischemic attack” OR tia OR poststroke OR “cerebrovascular accident” OR cva OR “brain attach hemorrhage” |
| #2 | rehabilitation OR exercise OR “rehabilitation therapy” OR physiotherapy OR “occupational therapy” |
| #3 | engage* |
| #4 | scale OR measure* OR questionnaire OR survey OR assessment OR monitor OR tool OR instrument |
| #5 | #1 AND #2 AND #3 AND #4 |

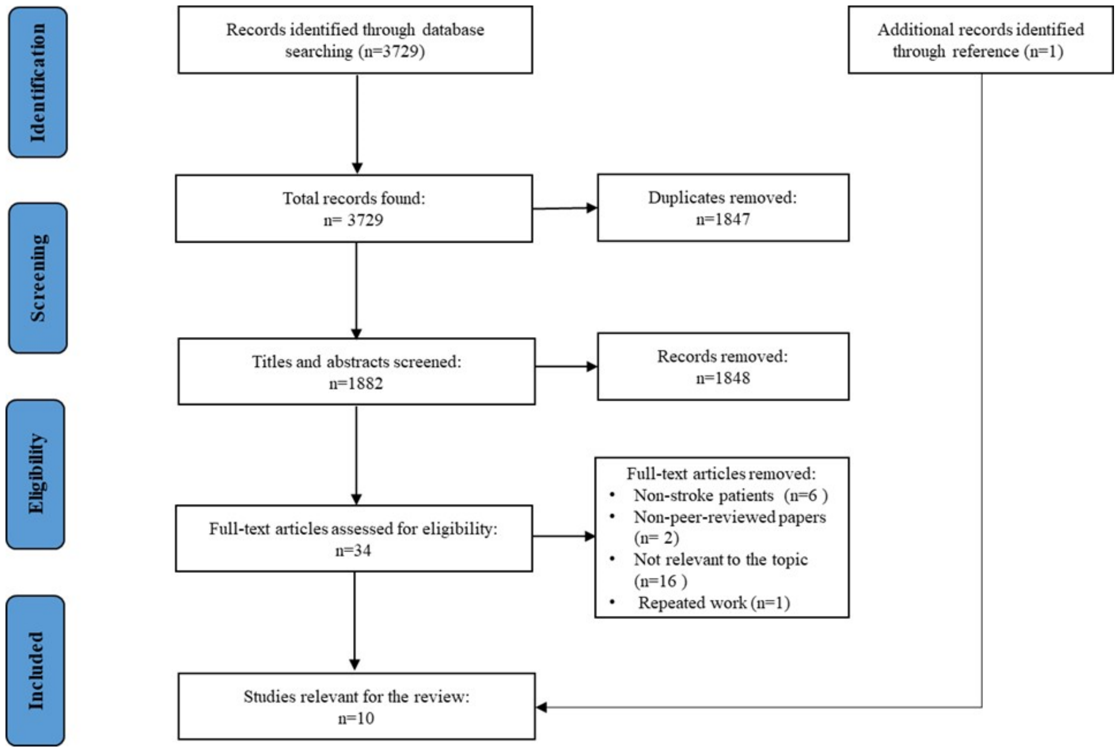


Figure S1. Literature screening flow diagram.
